# 734. Trend Analysis of Oritavancin and Comparator Agents Activity against Enterococcus Causing Infections in US Medical Centers between 2017–2019 and 2022

**DOI:** 10.1093/ofid/ofad500.795

**Published:** 2023-11-27

**Authors:** Cecilia G Carvalhaes, Rodrigo E Mendes, Dee Shortridge, Mariana Castanheira

**Affiliations:** JMI Laboratories, North Liberty, IA; JMI Laboratories, North Liberty, IA; JMI Laboratories, North Liberty, IA; JMI Laboratories, North Liberty, IA

## Abstract

**Background:**

A new formulation of oritavancin (ORI) to be infused over 1 hour for the treatment of skin and skin structure infections was approved in 2021 by the US FDA. The activity of ORI and its comparators against *Enterococcus* (ENT) and resistant (R) subsets from US medical centers was evaluated comparatively between 2022 and 2017**–**2019.

**Figure:**
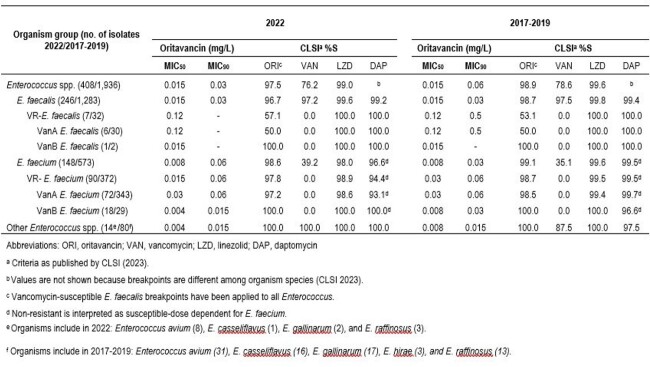

**Methods:**

4,462 ENT, including 246/2,556 *E. faecalis* (EF) from 2022 and 2017**–**2019, respectively, 148/1,348 *E. faecium* (EFM), and 14/150 other ENT were collected (1/patient) from 34 US medical centers. Isolates were identified by MALDI-TOF MS and standard microbiology tests and susceptibility (S) tested by CLSI broth microdilution. CLSI clinical breakpoints (BPs) and VanA/VanB phenotypes were used. ORI BPs against vancomycin (VAN)-S EF were applied to all ENT.

**Results:**

ORI activity against ENT from 2022 (MIC_50/90_, 0.015/0.03 mg/L) was similar to 2017**–**2019 (MIC_50/90_, 0.015/0.06 mg/L; Table). ORI inhibited 97.5%/98.9% of ENT from 2022/2017**–**2019 at ≤ 0.12 mg/L. VAN and linezolid (LZD) inhibited 97.2%/98.4% and 99.6%/99.6% of ENT from 2022/2017**–**2019, at the respective BPs. ORI, VAN, LZD and daptomycin (DAP) showed stable S rates ( > 96%) against EF. ORI remained active against 57.1/53.1% of VAN-R EF. VanA rates in VAN-R EF from 2022 and 2017**–**2019 were 85.7%/93.8%. ORI inhibited the 3 VanB EF isolates at ≤ 0.03 mg/L. LZD and DAP remained active against VAN-R EF (100%). Similar activity was noted for ORI against EFM from 2022 (MIC_50/90_, 0.008/0.06 mg/L) and 2017**–**2019 (MIC_50/90_, 0.008/0.03 mg/L). The EFM S rate to VAN was 35.1% in 2017**–**2019 and 39.2% in 2022. The S rates to ORI (VAN-S EF BPs) and LZD remained stable ( > 98%) as well as rates of DAP susceptible dose-dependent (SDD; 96.6% in 2022 and 99.5% in 2017**–**2019). The VanB phenotype increased from 7.8% in 2017**–**2019 to 20.0% in 2022. ORI inhibited all VanB and 98.5% of VanA EFM at ≤ 0.12 mg/L. LZD inhibited 98.6%/99.4% of VanA EFM isolates in 2022/2017**–**2022. The DAP SDD rate slightly decreased against VanA EFM (99.7% to 93.1%). ORI and comparators were active against other ENT.

**Conclusion:**

ORI exhibited potent and stable activity against ENT clinical isolates, including VAN-R EFM in US. An increase in VanB EFM phenotype and a slight decrease in the DAP SDD rates in VAN-R EFM subsets were noted over time.

**Disclosures:**

**Cecilia G. Carvalhaes, MD, PhD**, AbbVie: Grant/Research Support|bioMerieux: Grant/Research Support|Cipla: Grant/Research Support|CorMedix: Grant/Research Support|Melinta: Grant/Research Support|Pfizer: Grant/Research Support **Rodrigo E. Mendes, PhD**, AbbVie: Grant/Research Support|Basilea: Grant/Research Support|Cipla: Grant/Research Support|Entasis: Grant/Research Support|GSK: Grant/Research Support|Paratek: Grant/Research Support|Pfizer: Grant/Research Support|Shionogi: Grant/Research Support **Dee Shortridge, PhD**, Melinta: Grant/Research Support|Shionogi: Grant/Research Support **Mariana Castanheira, PhD**, AbbVie: Grant/Research Support|Basilea: Grant/Research Support|bioMerieux: Grant/Research Support|Cipla: Grant/Research Support|CorMedix: Grant/Research Support|Entasis: Grant/Research Support|Melinta: Grant/Research Support|Paratek: Grant/Research Support|Pfizer: Grant/Research Support|Shionogi: Grant/Research Support

